# EdgeGeoDiff: A Novel Two-Stage Diffusion Approach for Precipitation Downscaling with Edge Details and Geographical Priors

**DOI:** 10.3390/s26061857

**Published:** 2026-03-15

**Authors:** Shiji Zhang, Chenghong Zhang, Tao Wu, Tao Zou, Yuanchang Dong

**Affiliations:** 1 School of Computer Science, Chengdu University of Information Technology, Chengdu 610225, China; 3230608024@stu.cuit.edu.cn; 2 Institute of Plateau Meteorology, China Meteorological Administration, Chengdu 610072, China; zhangchenghong@cma.gov.cn (C.Z.);; 3 Yibin Meteorological Bureau, Yibin 644000, China

**Keywords:** precipitation, super-resolution, downscaling, diffusion model, high-frequency component enhancement

## Abstract

Precipitation downscaling aims to enhance coarse-resolution data to higher resolutions. Due to the similarity between downscaling and super-resolution (SR), deep learning-based SR approaches have been increasingly adopted in this domain. However, single-image super-resolution (SISR) methods applied to precipitation data face two main challenges: weak high-frequency signals and highly skewed distributions in precipitation datasets, which often lead to overly smooth reconstructions, failure to capture precipitation extremes, and loss of fine-scale variability with predictions biased toward mean values. To address these issues, we propose EdgeGeoDiff, a two-stage diffusion model for precipitation downscaling that leverages both edge information and geographical priors (e.g., terrain-related factors such as elevation). In the first stage, a residual network reconstructs an initial high-resolution precipitation field with preliminary structural details. In the second stage, edge features extracted using the Laplacian operator, together with geographical priors, guide a diffusion model to generate residuals that enhance fine-scale precipitation structures. Experimental results on real-world precipitation datasets show that EdgeGeoDiff effectively reconstructs fine-scale details while preserving large-scale patterns and outperforms conventional SISR methods in terms of its RMSE, PSNR, SSIM, and CSI, particularly demonstrating superior performance in the high-frequency region of the spectrum.

## 1. Introduction

Precipitation is a fundamental component of the Earth’s climate system, with a significant impact on ecological, climatological, and hydrological processes, making it a critical focus in related scientific research. Accurate simulation and prediction of precipitation patterns at local scales is vital to understanding the impacts of climate change, particularly in regions vulnerable to extreme weather events such as droughts, floods, and storms [1]. However, the pixel resolution of precipitation datasets is usually too coarse—often to the order of 10–25 km—to be applied effectively in hydrological and meteorological studies, especially at local or regional scales. In recent years, several studies have focused on improving the resolution of precipitation estimates and proposed numerous algorithms.

Downscaling refers to the process of transforming coarse-resolution climate or meteorological data into finer spatial resolutions while preserving the underlying physical patterns. This problem is particularly relevant for precipitation datasets, where observations from satellites or radar systems often provide large-scale coverage but lack sufficient spatial detail for regional analysis. In this study, we perform precipitation downscaling using paired low-resolution and high-resolution precipitation fields from real-world observational datasets. By learning the mapping from coarse to fine spatial scales, downscaling methods aim to reconstruct detailed precipitation structures that are crucial for understanding local weather variability, extreme precipitation events, and hydrological processes.

Downscaling techniques mainly include dynamical downscaling [2], statistical downscaling [3], and methods based on deep learning. Dynamic downscaling uses regional climate models (RCMs) nested within global climate models (GCMs) to generate high-resolution climate information. This approach relies on the detailed mathematical representation of complex physical processes in the oceans, atmosphere, and land surfaces. For instance, while it offers more precise, localized climate projections, dynamical downscaling requires substantial computational resources and large volumes of data, which can limit its practical applicability [4]. Statistical downscaling discovers and establishes empirical relationships between large-scale variables (e.g., atmospheric circulation, temperature, humidity, and wind fields derived from GCMs) and local variables (e.g., station-scale precipitation or surface observations) and then applies these relationships to the outputs of regional or global models. In addition to precipitation pairing datasets, statistical downscaling requires high-resolution data with matched spatio-temporal dimensions, such as wind fields, temperature fields, and geographical information, to establish more accurate empirical statistical relationships [5]. This approach faces challenges in regions with complex topography or unique climate zones, as the quality of the input data significantly impacts the accuracy of the results.

The tasks of downscaling and super-resolution (SR) share significant methodological similarities, as deep learning-based downscaling methods are primarily derived from SR techniques in the field of computer vision. Mainstream SR techniques are mainly based on convolutional neural networks (CNNs) [5], generative adversarial networks (GANs) [6], and vision transformers [7]. The denoising diffusion probabilistic model (DDPM) [8] has shown remarkable performance in image synthesis and has subsequently been widely adopted for super-resolution application [9,10]. Compared with GAN-based methods, diffusion-based models offer a more stable training process, enabling diffusion-based models to effectively fit the underlying data distribution and produce high-quality super-resolution results.

These methods demonstrate strong performance on natural image SR datasets. However, compared with natural image datasets, precipitation datasets present two primary challenges. Although precipitation fields may contain localized sharp gradients associated with convective storms, many precipitation events are dominated by large-scale weather systems that produce relatively smooth spatial patterns and limited grid-scale variability [11]. This characteristic makes the reconstruction of fine-scale structures more difficult. Second, precipitation datasets typically exhibit highly skewed distributions, where most grid values correspond to low or zero precipitation while extreme events occur infrequently. This skewed characteristic may complicate the learning process of the underlying models and cause the model to focus disproportionately on extreme cases. Consequently, conventional SISR methods may either insufficiently capture fine-scale spatial structures or exhibit biased learning toward extreme precipitation events.

**Figure 1:**
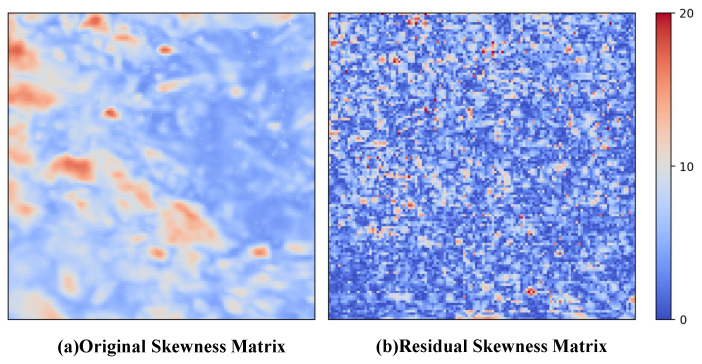
Construction of skewness matrices based on the skewness coefficients of original data (**a**) and residuals (**b**) across grid cells in the entire dataset. The skewness matrix of the residuals is processed using absolute value transformation.

To address the aforementioned challenges, we adopt a diffusion-based residual framework to better model the differences between the initial prediction and the ground truth. Instead of directly learning the highly skewed precipitation distribution, the diffusion model focuses on learning the residual spatial structures, which simplifies the learning objective under skewed precipitation distributions. As shown in Figure 1, this framework improves the ability of the diffusion model to reconstruct fine-scale precipitation details. Inspired by [12,13], in the first stage, we employ a Preceding High-Frequency Augmentation Network (Pre-HFAN) to generate an initial high-resolution output and compute the residual between this output and the high-resolution ground truth. This residual, which represents the high-frequency components of the precipitation data, is then used as the target for the diffusion model in the second stage. For the second stage, since topography is closely related to precipitation patterns and significantly influences the high-frequency components, we use both the initial high-resolution output and topography information as guiding conditions for the diffusion model to further enhance the high-frequency components of the initial output.

To summarize, our contributions are as follows.

- We propose a two-stage downscaling framework for high-frequency component enhancement, where geographical information is also used as a guiding condition to improve the realism of the high-resolution output.
- Our method employs a residual structure that effectively mitigates the skewness in the precipitation dataset, thereby enhancing the model’s ability to recover both low-frequency and high-frequency components.
- We introduce two modules to enhance the diffusion model’s awareness of additional guiding conditions, including edge details and geographical priors.

## 2. Related Work

### 2.1. Single-Image Super-Resolution

Single-image super-resolution (SISR) is a technique that generates high-resolution (HR) images from low-resolution (LR) counterparts that is widely applied in the field of computer vision. The SRCNN [14] was the first convolutional neural network proposed for mapping LR images to HR images. However, the SRCNN suffers from limited representational capacity and relies on external upsampling, which constrains its ability to recover fine high-frequency details. The DRCN [15] leverages a recursive architecture to enhance the performance of convolutional networks by reusing features across multiple layers. The DRCN achieves deeper modeling via recursive layers but faces training instability and limited expressive power due to parameter sharing, hindering high-quality super-resolution. Subsequently, numerous CNN-based methods have been proposed, which enhance super-resolution performance by modifying network architectures or adjusting loss functions [16,17,18]. Although these CNN-based methods improve pixel-wise accuracy, they are limited in recovering fine textures or high-frequency details due to shallow or recursive network structures, reliance on MSE loss, and insufficient modeling of complex image statistics. To address these limitations, the SRGAN [19] was proposed, leveraging a GAN framework to produce sharper, more realistic images with enhanced texture details. The ESRGAN [20] refined the network architecture and loss functions, further improving the generative quality of the SRGAN. The GAN-based generative model effectively mitigates transition smoothing and produces improved perceptual quality [21,22]. But GAN-based methods are prone to mode collapse [23], where the generator produces limited diversity despite varied input. Furthermore, the GAN-based training process is difficult to converge and requires an additional discriminator, which is not used during inference [19].

### 2.2. Diffusion-Based SR Methods

The DDPM has demonstrated strong performance in the field of image synthesis [24,25] and has garnered widespread attention in the super-resolution domain due to its ability to generate high perceptual quality results with improved diversity. SrDiff [26] is the first diffusion-based model applied to SISR, demonstrating the applicability of diffusion models in the super-resolution domain. Sr3 [10], based on the DDPM, uses the results of interpolated up-sampling as guidance to achieve high perceptual quality in the mapping from LR to HR. ResDiff [12] employs a simple convolutional neural network followed by frequency domain information enhancement to further refine the super-resolution results and achieve high-quality generation outcomes.

Compared with previous methods, our approach places greater emphasis on the recovery of high-frequency components, employing a two-stage high-frequency enhancement strategy which utilizes edge detection-based high-frequency information and geographical data to guide the generation of high-resolution precipitation data.

## 3. Materials and Methods

### 3.1. Pre-HFAN

#### 3.1.1. Network Stucture

**Figure 2:**
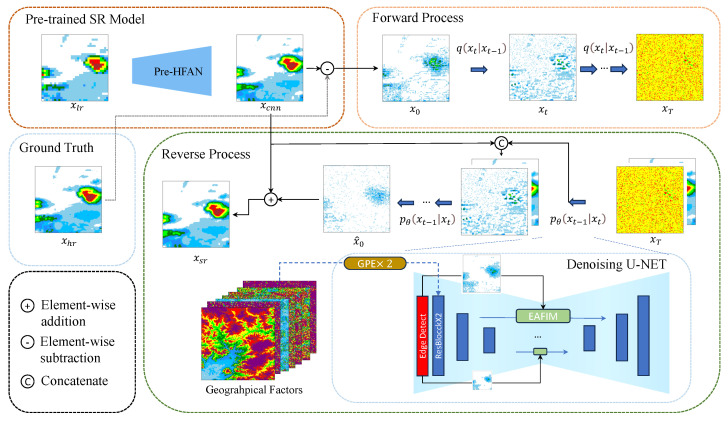
The proposed method utilizes the pretrained pre-HFAN for initial super-resolution reconstruction. The residual between the $x_{hr}$ and $x_{cnn}$ results is used as the target for the diffusion model. The input to the denoising U-Net consists of $x_{cnn}$, $x_{t}$, geographical factors, and the edge map of $x_{cnn}$, which are mapped to different scales and fused with original features through the EAFIM. The geographical factors is introduced via GPE.

As shown in Figure 2, the goal of our method is to generate an initial high-resolution result with strong high-frequency components, which will serve as guiding conditions for the diffusion model in the next stage. To achieve this, we introduce the Pre-HF Augmentation Network (Pre-HFAN), which is designed to enhance the high-frequency components in the generated result before feeding them into the diffusion model. Meanwhile, to maintain the model’s time efficiency, a simple neural network architecture is used, consisting of only a few convolutional and ReLU activation layers. The convolutional layer employs central difference convolution [27] to enhance the perception of intrinsic detailed patterns. The final upsampling step is carried out using pixel shuffle [28]. We employ a residual connection from input to output, where the network parameters actually predict the residual between the output and the low-resolution interpolated result. The interpolated result contains almost no high-frequency information, while the shallow convolutional network is able to capture high-frequency components and supplement the interpolated result with additional high-frequency details.

#### 3.1.2. Loss Function

Edge-based and frequency-domain analyses are widely applied in the fields of image reconstruction [13,29] and image segmentation [30]. As mentioned above, the output of the Pre-HFAN is the residual between the generated high-resolution result and the interpolated result. A pixel-wise loss function may lead to excessive smoothing and is also susceptible to the influence of precipitation skewness. Thus, we adopted a high-frequency augmentation loss function, which focuses on maintaining the global structure and enhancing high-frequency details. The fast Fourier transform (FFT) transforms a time-domain signal (grid data) into the frequency domain while preserving the spatial dimensions. Each grid contains the full information of the signal in the time domain. Therefore, we use an FFT-based loss function (FFTLoss) [12] to maintain the overall structure of the initial high-resolution result. The calculation is as follows:

(1) $$\begin{array}{c} Loss_{FFT}={∥F\left(x_{cnn}\right)-F\left(x_{hr}\right)∥}_{2}^{2} \end{array}$$

where F(·) denotes the 2D fast Fourier transform (FFT), $x_{cnn}$ represents the output of the Pre-HFAN, and $x_{hr}$ refers to the high-resolution ground truth.

For precipitation data at small scales, the numerical values typically do not experience drastic changes between grids, meaning that high-frequency components are relatively sparse compared with natural images. However, high-frequency components are crucial for assessing the quality of super-resolution reconstruction. Therefore, we adopted $Loss_{EDGE}$ [13], based on edge detection, and $Loss_{DWT}$ [12], based on the discrete wavelet transform (DWT), to enhance the high-frequency components of the output generated by the Pre-HFAN.

To emphasize structural fidelity, we incorporate HAAR wavelet decomposition into the loss function. Specifically, the image is decomposed into low- and high-frequency sub-bands using Haar wavelets. The calculation of $Loss_{DWT}$ can be described as follows:

(2) $$Loss_{DWT}=\sum_{i=l}^{L}\left[{\left|\left|H_{i}-{\hat{H}}_{i}\right|\right|}_{2}^{2}+{\left|\left|V_{i}-{\hat{V}}_{i}\right|\right|}_{2}^{2}+{\left|\left|D_{i}-{\hat{D}}_{i}\right|\right|}_{2}^{2}\right]$$

where $H_{i}$, $V_{i}$, and $D_{i}$ are the sub-bands of the ground truth in the *i*th level of the DWT and ${\hat{H}}_{i}$, ${\hat{V}}_{i}$ and ${\hat{D}}_{i}$ are the sub-bands of the Pre-HFAN output in the *i*th level of the DWT, while *L* is the total DWT level count.

In image processing, edge detection and high-frequency components are intrinsically linked. This relationship arises because edges are typically characterized by sharp transitions in pixel values, which manifest as rapid changes in the frequency spectrum. Therefore, we employ an edge detection-based loss function to enhance the high-frequency components of the generated image. The calculation of $Loss_{EDGE}$ can be described as follows:

(3) $$\begin{array}{c} Loss_{EDGE}=\sqrt{{∥\Delta \left(x_{cnn}\right)-\Delta \left(x_{hr}\right)∥}_{2}^{2}+\epsilon^{2}} \end{array}$$

where Δ denotes the Laplacian operator and ϵ represents a predefined constant value used to mitigate the vanishing gradient problem. The final loss function is described as follows:

(4) $$\begin{array}{c} Loss_{HFA}=\alpha Loss_{FFT}+\beta Loss_{DWT}+\gamma Loss_{EDGE} \end{array}$$

where α, β, and γ are tunable hyperparameters of the loss function. The results of the diffusion model generation are influenced by its guiding conditions. Specifically, in our approach, the diffusion model generates the residual between the ground truth (GT) and the output of the Pre-HFAN. In this process, the diffusion model focuses on generating high-frequency components, making it more dependent on the high-frequency guidance provided by the output of the Pre-HFAN.

### 3.2. EG-Guided Diffusion

After obtaining the output of the Pre-HFAN, we employ a diffusion model for further refinement, generating results with finer details. The predictions of the diffusion model represent the residual between the ground truth and the output of the Pre-HFAN, which serves to complement the high-frequency components of the Pre-HFAN output.

#### 3.2.1. Conditional Diffusion Model

The diffusion model [8] consists of two main components: the forward process and the reverse process. The forward process maps discrete data to a continuous noise distribution. Following Sr3 [10], in the forward process, the input residual $x_{0}$ is iteratively corrupted with noise, transforming it into an approximated noise $x_{T}$. The forward process of diffusion model can be expressed as follows:

(5) $$\begin{array}{c} q\left(x_{t}|x_{t-1}\right)=N\left(x_{t};\sqrt{1-\beta_{t}}x_{t-1},\beta_{t}I\right) \end{array}$$

where N(·) denotes a Gaussian (normal) distribution, $\beta_{t}$ represents the level of noise added to the data at time step *t*, and *t* is sampled from p(T). The specific iterative noise addition process is as follows:

(6) $$\begin{array}{c} x_{t}=\sqrt{{\bar{\alpha }}_{t}}x_{0}+\left(1-{\bar{\alpha }}_{t}\right)\epsilon \end{array}$$

where $\alpha_{t}=1-\beta_{t}$ controls the proportion of the original signal preserved at step *t*, while ${\bar{\alpha }}_{t}=\prod_{i=0}^{t}\alpha_{i}$ represents the cumulative signal retention over the diffusion process. In the reverse process, the diffusion model samples from the noise distribution and, under conditional guidance, iteratively removes noise corresponding to each noise level to generate the predicted residual ${\hat{x}}_{0}$ The reverse process can be described as follows:

(7) $$\begin{array}{c} x_{t-1}=\frac{1}{\alpha_{t}}\left(x_{t}-\frac{\beta_{t}}{1-{\bar{\alpha }}_{t}}\epsilon_{\theta }\right)+\sqrt{\frac{1-{\bar{\alpha }}_{t-1}}{1-{\bar{\alpha }}_{t}}\beta_{t}} \end{array}$$

where $\epsilon_{\theta }$ represents the predicted noise at time step *t* and is estimated using a U-Net architecture, while ϵ denotes the randomly sampled noise. The model iteratively denoises the data to generate the predicted residual ${\hat{x}}_{0}$. For model optimization, the target for training $\epsilon_{\theta }$ is as follows:

(8) $$\begin{array}{c} \min_{\theta }L\left(\theta \right)=E_{\epsilon ,x_{t}}[\|\epsilon -\epsilon_{\theta }\left(x_{t},t,x_{cnn},c_{N}\right){\|}_{1}] \end{array}$$

where θ denotes the learnable parameters of the diffusion network, ϵ is the randomly sampled Gaussian noise, $\epsilon_{\theta }\left(\cdot \right)$ denotes the predicted noise, $x_{t}$ represents the noisy sample at diffusion step *t*, and $c_{N}$ denotes the set of geographical factors used as conditional inputs, where *N* is the number of terrain variables.

#### 3.2.2. Geographical Prior Encoder

**Figure 3:**
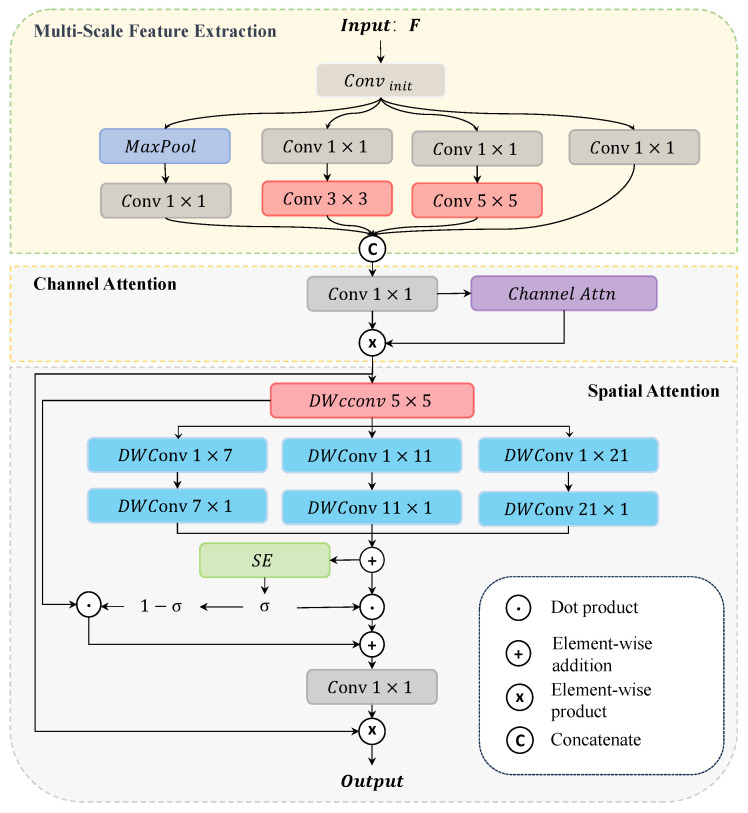
Geographical prior encoder (GPE).

Geographical factors, such as the elevation, slope, and aspect, are closely related to precipitation formation and distribution, significantly influencing the high-frequency components of local precipitation. Geographical factors encompass not only characteristics associated with precipitation distribution but also a substantial amount of redundant information. As shown in Figure 3, given an intermediate feature map $F_{0}\in R^{C\times II\times W}$ as input, the computation of the initial multi-scale features can be described as follows:

(9) $$\begin{array}{cc} F_{s}=concat & (Swish(Conv_{3\times 3}\left(LR\left(Conv_{1\times 1}\left(F_{0}\right)\right)\right)),\\ & Swish(Conv_{5\times 5}\left(LR\left(Conv_{1\times 1}\left(F_{0}\right)\right)\right)),\\ & Swish(Conv_{1\times 1}\left(MaxPool\left(F_{0}\right)\right)),\\ & Swish(Conv_{1\times 1}\left(F_{0}\right)) \end{array}$$

where $Conv_{k\times k}$ represents k×k convolution that maintains the number of channels. Conversely, $Conv_{1\times 1}$ represents 1×1 convolution that reduces the number of channels to 1/4 of the original. LR(·) refers to the Leaky ReLU activation function, while Swish(·) denotes the Swish activation function. Inspired by [31], we further enhanced these features by integrating spatial attention that incorporates channel information. This approach allows us to effectively emphasize significant features while suppressing irrelevant noise. Following the method proposed by CBAM [32], the calculation of the channel attention can be described as follows:

$$\begin{array}{c} CA=Sigmoid(MLP\left(AvgPool\left(F_{s}\right)\right)+MLP\left(MaxPool\left(F_{s}\right)\right)) \end{array}$$

(10) $$\begin{array}{c} F_{c}=F_{s}⊗CA \end{array}$$

where ⊗ denotes element-wise multiplication, MLP(·) denotes a multi-layer perceptron, and Sigmoid(·) represents the sigmoid function. To capture spatial dependencies and global information, we employed large kernel depth-wise convolutions. The computation can be described as follows:

(11) $$\begin{array}{c} F_{i}=DwConv_{k\times 1}\left(DwConv_{1\times k}\left(DWConv_{5\times 5}\left(F_{c}\right)\right)\right),\;\;\;k\in \left\{7,11,21\right\} \end{array}$$

where DwConv represents depth-wise convolution, i ϵ [0,1,2] represents a different branch, and *k* denotes the kernel size of the asymmetric depthwise convolutions, which takes values from {7,11,21} to construct three parallel branches. This structure follows MSCA [33]. Furthermore, we applied additional weighting to both the spatial and channel feature maps to improve the effectiveness of feature fusion, thereby generating the spatial attention map. The computation can be described as follows:

(12) $$\begin{array}{cc} & \rho =Sigmoid(AvgPool\left(ResSE\left(F_{1}+F_{2}+F_{3}\right)\right))\\ & SA=Conv_{1\times 1}\left(\rho ⊗\left(F_{1}+F_{2}+F_{3}\right)+\left(1-\rho \right)⊗F_{c}\right)\\ & output=SA⊗F_{c} \end{array}$$

where ResSE represents the residual squeeze-and-excitation block [12] and ρ denotes a spatial weight map. ResSE focuses on channel information, while the average pooling operation emphasizes spatial information, collectively generating the weight coefficients.

#### 3.2.3. Edge-Aware Feature Integration Module

**Figure 4:**
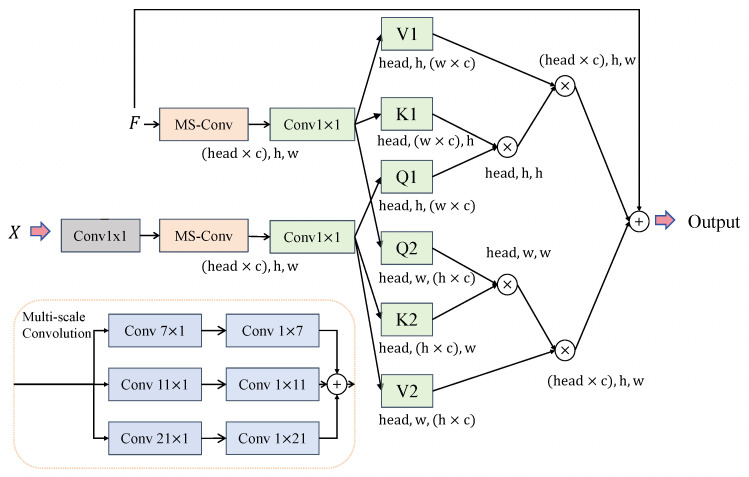
Edge-Aware Feature Integration Module (EAFIM).

The generation results of the diffusion models are influenced by the guiding conditions. The original U-Net architecture concatenates the features of the encoder and decoder, which limits its ability to perceive high-frequency components effectively. Inspired by [30], we propose the Edge-Aware Feature Integration Module (EAFIM) to enhance residual recovery in diffusion models by incorporating structural priors. As shown in Figure 4, this module processes the edge maps already downsampled from $x_{cnn}$ Laplacian edge detection to obtain the edge features at multiple scales, allowing mutual querying with the corresponding features of the encoder at each scale. For the input feature map $F\in R^{C\times H\times W}$ and the edge map $X\in R^{1\times H\times W}$, we define the calculation of *Q*, *K*, and *V* as follows:

(13) $$\begin{array}{cc} Q,K,V= & Conv_{1\times 1}(DWConv_{7}\left(GN\left(F\right)\right)\\ & \;+DWConv_{11}\left(GN\left(F\right)\right)\\ & \;+DWConv_{21}\left(GN\left(F\right)\right)) \end{array}$$

where GN(·) represents group normalization and $DWConv_{i}$ represents depth-wise convolution with a kernel size of 1× i and i× 1. $F_{e}\in R^{C\times H\times W}$ denotes the edge map *X* processed through a convolutional layer to match the channel dimension of *F*, followed by multi-scale convolutions to compute $Q_{1}$, $K_{1}$, and $V_{1}$. After obtaining the $Q_{1}$, $K_{1}$, and $V_{1}$ from $F_{e}$ and $Q_{2}$, $K_{2}$, and $V_{2}$ from *F*, we calculate their cross-attention, with the attention formula being

(14) $$\begin{array}{c} Attn\left(Q,K,V\right)=Softmax\left(\frac{QK^{T}}{\sqrt{d_{k}}}\right)V \end{array}$$

where $d_{k}$ is the number of columns in matrix Q. Then, the two feature maps perform cross-attention computation by querying each other. The steps of the calculation are as follows:

(15) $$\begin{array}{cc} & F_{1}=Conv_{1\times 1}\left(Attn\left(Q2,K1,V1\right)+Q2\right)\\ & F_{2}=Conv_{1\times 1}\left(Attn\left(Q1,K2,V2\right)+Q1\right) \end{array}$$

where $F_{1}$ and $F_{2}$ combine both edge features and original features, the final output of the model is given by

(16) $$\begin{array}{c} F_{out}=F_{1}+F_{2}+F \end{array}$$

$F_{out}$ will be fused with the deeper features through a skip connection.

### 3.3. Metrics

Applied to single-channel precipitation data, we evaluated the model’s performance by employing several image quality metrics, including the peak signal-to-noise ratio (PSNR) [34], structural similarity index (SSIM) [34], and root mean square error (RMSE) [35]. To evaluate the model’s ability to recover high-frequency components, we compute the mean squared error (MSE) of the radially averaged power spectral density (PSD) curves [36], as well as the multi-band structural similarity index (SSIM) and the critical success index (CSI) [37]:

(17) $$\mathrm{PSNR}=10\cdot \log_{10}\left(\frac{L^{2}}{\mathrm{MSE}}\right)$$

where *L* is the maximum possible pixel value and MSE is the mean squared error between the predicted and ground-truth images:

(18) $$\mathrm{MSE}=\frac{1}{N}\sum_{i=1}^{N}{\left(x_{i}-y_{i}\right)}^{2}$$

(19) $$\mathrm{RMSE}=\sqrt{\frac{1}{N}\sum_{i=1}^{N}{\left(x_{i}-y_{i}\right)}^{2}}$$

where $x_{i}$ and $y_{i}$ represent the pixel intensities of the predicted and ground-truth images, respectively, and *N* is the total number of pixels:

(20) $$\mathrm{SSIM}\left(x,y\right)=\frac{\left(2\mu_{x}\mu_{y}+C_{1}\right)\left(2\sigma_{xy}+C_{2}\right)}{\left(\mu_{x}^{2}+\mu_{y}^{2}+C_{1}\right)\left(\sigma_{x}^{2}+\sigma_{y}^{2}+C_{2}\right)}$$

where $\mu_{x}$ and $\mu_{y}$ are the local means, $\sigma_{x}^{2}$ and $\sigma_{y}^{2}$ are the local variances, and $\sigma_{xy}$ is the local covariance of the predicted image *x* and ground-truth image *y*. Constants $C_{1}$ and $C_{2}$ are used to stabilize the division:

(21) $$\mathrm{PSD}-\mathrm{MSE}=\frac{1}{N}\sum_{i=1}^{N}{\left(P_{\mathrm{pred}}\left(i\right)-P_{\mathrm{gt}}\left(i\right)\right)}^{2}$$

where $P_{\mathrm{pred}}\left(i\right)$ and $P_{\mathrm{gt}}\left(i\right)$ denote the normalized, radially averaged power spectral density (PSD) values at the *i*th frequency bin for the predicted and ground-truth images, respectively, and *N* is the total number of radial bins:

(22) $$\mathrm{CSI}=\frac{\mathrm{TP}}{\mathrm{TP}+\mathrm{FN}+\mathrm{FP}}$$

where TP is the number of true positives (correctly predicted precipitation), FP is the number of false positives (non-precipitation predicted as precipitation), and FN is the number of false negatives (missed precipitation events).

## 4. Results

### 4.1. Dataset

The dataset included high-resolution hourly precipitation data for the North China region from 2021 to 2023, comprising a total of 53 weather event datasets and 19,000 files. The spatial coverage was between 35.8°–42.8° N and 112.8°–120° E, with a grid resolution of 1400 × 1440. The specific data consisted of hourly precipitation (mm/h) with a spatial resolution of 500 m, while lower-resolution data were obtained through upscale processing. In this paper, we used a paired dataset with resolutions ranging from 3000 m to 750 m. The dataset was cropped to the spatial range of 40.06°–41.02° N and 116.76°–117.72° E, corresponding to a grid size of 128 × 128. Regions without precipitation or only trace amounts were excluded, resulting in a total of 9640 data points, and we yielded 9640 samples split into 9290 training, 11 validation, and 339 test samples.

Geographical factors included elevation, slope, slope gradient, aspect, aspect gradient, topographic relief, and surface roughness. The spatial extent of these geographical data was consistent with that of the precipitation dataset.

### 4.2. Implementation Details

#### 4.2.1. Pre-HFAN

The model employed a three-layer central difference convolution, with the channel sizes of the feature map set to 64, 32, and 16, followed by upsampling using pixel shuffle. The pretraining was performed for 200 epochs with a batch size of 128, a learning rate of 10^−4^, and the Adam optimizer. The hyperparameters of the loss function, α, β, and γ, control the contributions of different loss components. Specifically, α is for the frequency-domain loss $Loss_{FFT}$ to enforce global frequency consistency between the reconstructed and high-resolution images, β is for the wavelet-domain loss $Loss_{DWT}$ to preserve multi-scale high-frequency details, and γ is for the edge-preserving loss $Loss_{EDGE}$ to enhance edge structures and maintain spatial sharpness. These coefficients were set to 1, 0.5, and 0.2 in our experiments, while the constant coefficient ϵ in $Loss_{EDGE}$ was set to 0.001 to ensure numerical stability.

#### 4.2.2. EG-Guided Diffusion

The input to the U-NET, $x_{t}$ and $x_{cnn}$, undergoes edge detection using the Laplacian operator on $x_{cnn}$, after which the three components are concatenated along the channel dimension. The U-Net architecture of the diffusion model has an input channel size of 64, with channel multipliers of [1, 2, 4, 8, 8]. Each layer of the U-Net contains two residual blocks [38], with self-attention applied only to the last two layers and the middle layer. The diffusion hyperparameter time step was set to 1500, and the β schedule increased linearly from 10^−6^ to 10^−2^. The training settings included a batch size of eight, a dropout rate of 0.2, a learning rate of 10^−4^, a total of 500,000 iterations, and the Adam optimizer. Additionally, all models were trained using the PyTorch 2.4.1 toolkit on a Tesla V100 GPU.

For a fair comparison, we maintained the same β schedule for the diffusion model as used in ResDiff [12] and Sr3 [10]. Since the time step significantly affects the quality of the generated results, we also set the time step to 1500 for these models.

All deep learning models were trained and evaluated under identical data splits and preprocessing procedures to ensure a fair comparison. The input and output data were normalized consistently across all methods. Model inference was performed on the same test set without post-processing.

### 4.3. Performance Comparisons

We compared the effectiveness of our model against several SISR methods, including CNN-based approaches such as EDSR [16], GAN-based approaches such as SRGAN [19], and diffusion-based methods such as Sr3 [10] and ResDiff [12].

#### 4.3.1. Quantitative Results

The quantitative results are summarized in Table 1. From a pixel-wise perspective, EdgeGeoDiff achieved the highest PSNR and the lowest RMSE among all compared methods, indicating that the explicit enhancement of high-frequency components did not compromise the overall numerical stability or introduce excessive pixel-level errors. Its performance was comparable to and slightly better than diffusion-based baselines such as Sr3 and ResDiff, demonstrating that the incorporation of edge-aware and geographic guidance remained well controlled at the pixel level.

Note that the PSNR values reported in this study are higher than those typically observed in natural image super-resolution tasks. This difference mainly arises from the characteristics of the precipitation fields. Precipitation data generally exhibit smoother spatial structures and contain large regions with low or zero precipitation intensity, which reduce pixel-wise reconstruction errors and consequently lead to higher PSNR values.

Interpolation-based bicubic resampling yielded relatively stable pixel-wise statistics for certain metrics, which can be attributed to its inherently smooth reconstruction behavior. By strongly suppressing high-frequency variations and enforcing spatial continuity, bicubic interpolation preserves low-frequency background structures effectively. However, this smoothing effect limits its ability to represent fine-scale precipitation variability, resulting in a substantially lower PSNR and higher RMSE compared with learning-based approaches.

More importantly, EdgeGeoDiff exhibited a clear advantage in terms of CSI, achieving the highest score among all methods. This improvement indicates that the recovered high-frequency components contributed directly to more accurate identification of precipitation regions, particularly by sharpening event boundaries and improving the spatial delineation of rainfall areas. In contrast, methods such as the SRGAN, despite producing visually sharper textures, showed noticeably lower CSIs, suggesting that their apparent high-frequency details did not consistently correspond to physically meaningful precipitation structures.

To further evaluate the precipitation detection capability of different models under varying rainfall intensities, the CSI scores were calculated under multiple precipitation thresholds ranging from 0.1 to 10. As shown in Table 2, the proposed EdgeGeoDiff consistently achieved the highest CSI across all thresholds. In particular, the improvement became more evident at moderate and heavy rainfall thresholds (e.g., 5 and 10), indicating that the proposed method can better preserve strong precipitation structures and reduce missed detections. These results demonstrate that incorporating geographical factors and edge guidance enables the model to more accurately reconstruct precipitation patterns across a wide range of rainfall intensities.

In the frequency domain, ResDiff achieved the lowest PSD-MSE, reflecting its conservative reconstruction strategy and strong global spectral consistency. EdgeGeoDiff reported a higher PSD-MSE, which can be attributed to its more aggressive enhancement of edge and texture components, leading to stronger high-frequency responses and a moderate increase in spectral deviation. Nevertheless, this deviation translated into tangible gains in spatial detection performance, as evidenced by the superior CSI. Compared with Sr3 and ResDiff, which rely primarily on implicit diffusion priors, the explicit integration of edge information and geographic factors in EdgeGeoDiff introduce physically meaningful spatial constraints. These constraints enable the model to selectively enhance structure-related high-frequency information, achieving a more favorable balance between numerical accuracy, precipitation detection capability, and physically interpretable spatial structures.

#### 4.3.2. Qualitative Comparison

**Figure 5:**
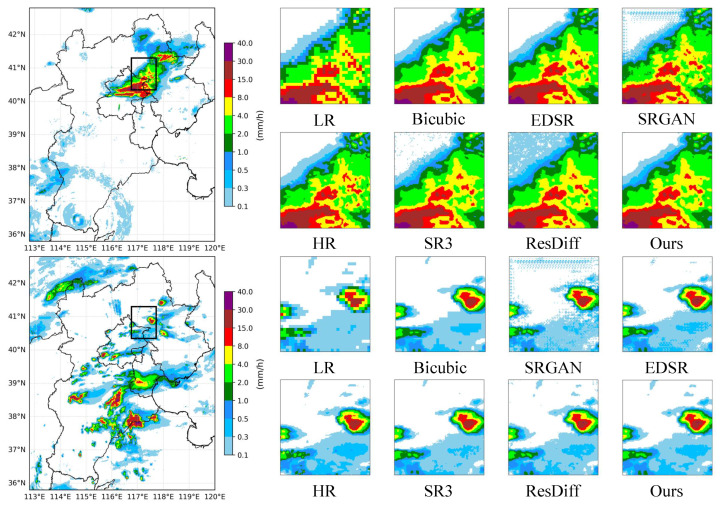
Visualization comparison of SR methods. The black box delineates the specific study area. The LR input has a resolution of 32 × 32 (corresponding to a 3000 m scale), while the HR reference has a resolution of 128 × 128 (corresponding to a 750 m scale). The second and fourth lines show the absolute differences between different models and the ground truth.

In Figure 5, Sr3, ResDiff, and the SRGAN all exhibited noticeable limitations in regions characterized by a high precipitation intensity. These methods tend to concentrate excessively on dominant rainfall cores while partially neglecting surrounding low-precipitation areas, resulting in blurred transitions and poorly defined precipitation boundaries. In particular, the SRGAN produced visually sharp textures with seemingly abundant high-frequency components; however, a closer inspection revealed that a substantial portion of these high-frequency patterns did not correspond to physically meaningful precipitation structures. Instead, they manifested as spurious fluctuations and artificial textures, which introduced additional errors and compromised spatial consistency.

The qualitative comparisons shown in the figure further highlight the superiority of the proposed method. In contrast to the aforementioned approaches, EdgeGeoDiff maintained stable and coherent reconstructions in low-precipitation regions, even when the overall precipitation intensity was high. Moreover, benefiting from the two-stage high-frequency enhancement strategy, the proposed method selectively reinforced structure-related high-frequency components, leading to clearer and more well-defined precipitation boundaries across a wide range of precipitation levels. As a result, EdgeGeoDiff provided a more accurate and visually interpretable representation of precipitation patterns, achieving superior performance in terms of both spatial stability and boundary clarity.

#### 4.3.3. High-Frequency Enhancement Evaluation

As shown in Table 3, EdgeGeoDiff consistently achieved the lowest PSD-MSE values across all high-frequency filtering levels, demonstrating its superior capability in recovering meaningful high-frequency components. This improvement became more pronounced as λ increased, indicating that the proposed method was particularly effective at reconstructing the mid-to-high-frequency structures associated with precipitation boundaries and localized intensity gradients.

It is worth noting that although the SRGAN exhibited visually prominent high-frequency patterns in qualitative comparisons, its quantitative PSD-MSE values remained substantially higher than those of the diffusion-based methods and EdgeGeoDiff, especially for smaller and intermediate λ values. This discrepancy suggests that the apparent high-frequency details generated by the SRGAN largely correspond to artificial textures and spurious fluctuations rather than physically meaningful precipitation structures. The elevated PSD-MSE values provide quantitative evidence that these visually sharp patterns do not align well with the true high-frequency spectral characteristics of the reference data.

Overall, EdgeGeoDiff demonstrated the best overall performance in terms of pixel-level fidelity and structural preservation. The moderate increase in spectral deviation reflects its enhanced capability in restoring the high-frequency component, suggesting strong potential for practical application in precipitation reconstruction tasks.

#### 4.3.4. Structural Consistency in High-Frequency Regions

To further evaluate the capability of different models in reconstructing precipitation structures across multiple spatial scales, we adopted a multi-band structural similarity metric (MB-SSIM) for frequency-domain evaluation. This metric decomposes precipitation fields into low-, mid-, and high-frequency components in the Fourier domain and computes the SSIM independently within each band. As a result, the MB-SSIM provides a frequency-dependent assessment of structural fidelity, enabling a more detailed analysis of how well large-scale coherence and fine-scale convective features are preserved.

**Figure 6:**
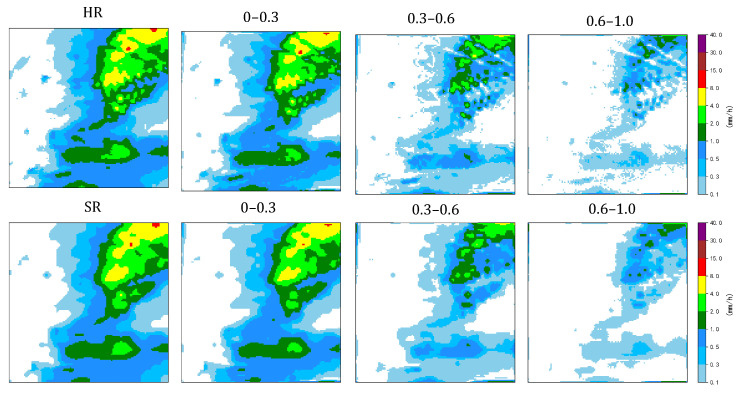
Visualization comparison of multi-band SSIM. HR denotes the ground truth with a resolution of 128 × 128, while SR represents the model output. The ranges of 0–0.3, 0.3–0.6, and 0.6–1.0 correspond to low-, mid-, and high-frequency components, respectively.

Specifically, normalized frequency-based spectral partitioning combined with Gaussian band-pass filtering was employed. The frequency domain was normalized to the range [0,1] according to the Euclidean distance from the spectral center and divided into three non-overlapping sub-bands: [0.0, 0.3], [0.3, 0.6], and [0.6, 1.0]. These bands approximately correspond to large-scale background patterns, mesoscale precipitation structures, and fine-scale spatial details, respectively, as illustrated in Figure 6.

The quantitative results are summarized in Table 4. The bicubic interpolation baseline consistently yielded the lowest SSIM values across all frequency bands, with particularly poor performance in the mid- and high-frequency ranges. This behavior reflects the inherent smoothing nature of interpolation-based methods, which preserve large-scale continuity at the expense of suppressing spatial variability and fine-scale precipitation structures.

Diffusion-based methods, including Sr3 and ResDiff, demonstrated noticeably improved structural similarity across all bands. ResDiff achieved the highest SSIM in the lowest frequency band, indicating strong capability in preserving large-scale precipitation coherence. However, its performance gradually decreased toward the higher frequency bands, suggesting limited effectiveness in reconstructing finer spatial details.

In contrast, EdgeGeoDiff attained the highest SSIM values in the high-frequency bands, indicating superior structural fidelity at the mesoscale and fine-scale levels. This advantage can be attributed to the explicit incorporation of edge information and frequency-aware guidance, which enables the model to selectively enhance structure-related high-frequency components while maintaining overall spatial consistency. Overall, these results demonstrate that EdgeGeoDiff achieved a more balanced multi-scale reconstruction, particularly excelling in the recovery of fine-scale precipitation structures that are critical for accurately representing spatial heterogeneity.

### 4.4. Ablation Study

In this section, we designed ablation experiments to demonstrate the effectiveness of each module. We tested the impact of different geographic factors and the residual structure on the generation results.

#### 4.4.1. Geographical Factor Impact

To investigate the contribution of geographic information to precipitation downscaling, we conducted a series of ablation experiments by progressively introducing different geographic factors into the model. Elevation was selected as the baseline factor due to its well-established physical relationship with precipitation formation. Additional factors were then incrementally incorporated, including the slope and slope gradient (three-factor), aspect and aspect gradient (five-factor), topographic relief (six-factor), surface roughness (six-factor*), and finally a comprehensive seven-factor configuration.

The quantitative results are reported in Table 5. Introducing elevation alone already led to a noticeable improvement over the configuration without geographic information, indicating that even a single physically meaningful factor can effectively guide the diffusion process. Expanding to the three-factor and five-factor configurations further improved the PSNR and CSI, suggesting that terrain orientation and local gradients provide complementary spatial cues that enhance the recovery of precipitation structures, particularly at finer scales.

Among all tested configurations, the five-factor set-up achieved the best overall performance across all evaluation metrics. This result indicates that the elevation, slope, and aspect-related variables jointly offer a balanced and informative representation of orographic influences on precipitation, enabling the model to better reconstruct spatial heterogeneity and sharp precipitation boundaries.

In contrast, incorporating additional factors such as topographic relief and surface roughness did not yield further performance gains. Instead, these configurations exhibited a degradation in PSNR and CSI. This behavior suggests that overly complex or redundant geographic inputs may introduce noise or conflicting signals into the diffusion process, thereby weakening its ability to accurately recover high-frequency precipitation components. Overall, these results highlight that the effectiveness of geographic conditioning depends not only on the quantity of factors but also on their physical relevance and mutual compatibility.

#### 4.4.2. Correlation Analysis

To further examine the relationship between geographical factors and the fine-scale precipitation structures reconstructed by the model, we conducted a statistical analysis using a random forest regression model. In this analysis, the precipitation residual field was used as the target variable. The residual is defined as the difference between the ground-truth high-resolution precipitation and the initial super resolution result produced by the pre-HFAN network. Seven geographical factors were used as predictors to evaluate their statistical relationship with the residual precipitation patterns.

The feature importance scores derived from the random forest model are reported in Table 6. Among all variables, elevation showed the largest contribution, followed by the aspect and slope gradient. This result indicates that both terrain elevation and orientation related factors provide useful information for modeling the residual precipitation structures that represent fine scale spatial variability.

Interestingly, the Pearson correlation coefficients between individual geographical factors and the precipitation residual field were close to zero. This observation suggests that the relationship between terrain characteristics and residual precipitation patterns is highly nonlinear and cannot be adequately described by simple linear correlations. In contrast, the random forest importance values revealed stronger nonlinear dependencies between geographical factors and the spatial distribution of precipitation residuals.

Notably, the geographical factors included in the best performing configuration in the ablation study, namely the five-factor setting, corresponded to those with relatively high nonlinear importance scores. This consistency further supports the effectiveness of incorporating physically meaningful geographical factors as conditioning information in the proposed diffusion based residual reconstruction framework.

#### 4.4.3. Effectiveness of Different Modules

The ablation results in Table 7 provide further insight into the roles of different modules in the proposed framework. With only the pre-HFAN module enabled, the model achieved a relatively high CSI while maintaining competitive PSNR and RMSE values. This behavior indicates that the baseline configuration produced smoother and more conservative precipitation fields, which reduce spurious responses near intensity thresholds and are therefore favorable for threshold-based detection metrics such as CSI.

When a GPE or EAFIM was introduced individually, both modules contributed to improved reconstruction accuracy in terms of the PSNR and RMSE, demonstrating their effectiveness in enhancing global spatial consistency and local structural representation, respectively. However, the CSI temporarily decreased in these intermediate configurations. This phenomenon can be attributed to the enhanced local gradients and sharper boundaries introduced by the additional modules, which may cause marginal precipitation regions to exceed the detection threshold and be counted as false alarms. As a result, the CSI did not monotonically increase with improved structural fidelity.

When both a GPE and EAFIM are jointly incorporated, the model achieved the best overall performance across all metrics, with simultaneous improvements in the PSNR, RMSE, and CSI. This result suggests that the two modules play complementary roles; the GPE imposes large-scale geographical constraints that suppress spatially inconsistent responses, while the EAFIM selectively reinforces structure-related high-frequency components. Their combined effect balances high-frequency enhancement and spatial coherence, effectively reducing false detections while preserving meaningful precipitation structures. Overall, the ablation study confirmed that integrating geographical priors and edge-aware mechanisms is essential for achieving robust high-frequency reconstruction and reliable precipitation detection in diffusion-based downscaling.

#### 4.4.4. Effectiveness of Residual Structure

To evaluate the effectiveness of the residual structure and its role in mitigating the skewness of the diffusion learning target, we conduct a comparative experiment between a non-residual formulation and the proposed residual design. In the non-residual setting, the diffusion model directly learns to map the low-resolution input to the high-resolution precipitation field, while in the residual formulation, the model predicts the residual between the input and the ground truth. All other hyperparameters and network configurations are kept identical to ensure a fair comparison.

As shown in Table 8, introducing the residual structure led to consistent improvements across all evaluation metrics. Specifically, the residual formulation improved the PSNR from 51.62 dB to 52.04 dB and reduced the RMSE from 0.333 to 0.312, indicating enhanced reconstruction accuracy. The corresponding increase in SSIM further suggests that the residual structure helps preserve structural coherence and fine-scale details.

**Figure 7:**
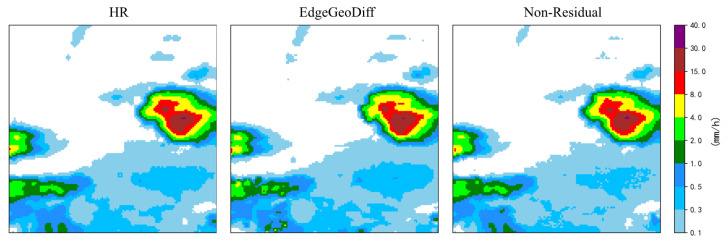
Visualization comparison of different structure.

As shown in Figure 7, the residual formulation exhibited clearer structural delineation and reduced background artifacts compared with the non-residual configuration, providing visual evidence for the improved quantitative performance.

These improvements can be attributed to the fact that residual learning reduces the dynamic range and distribution skewness of the diffusion target, allowing the model to focus on learning high-frequency corrections rather than reconstructing the entire precipitation field from scratch. As a result, the diffusion process becomes more stable and efficient, leading to better recovery of fine-scale structures and improved overall reconstruction quality.

## 5. Discussion

This study investigated diffusion-based precipitation downscaling from the perspective of high-frequency structure recovery rather than purely pixel-wise optimization. Unlike many existing super-resolution studies that primarily emphasize PSNR or SSIM improvements, our analysis highlighted the importance of frequency-domain consistency, boundary delineation, and detection-oriented performance for precipitation fields. These aspects are particularly relevant for meteorological applications, where spatial structure and event localization are often more critical than numerical smoothness.

The experimental results demonstrate that diffusion-based approaches, while not achieving state-of-the-art performance on conventional super-resolution benchmarks, exhibited notable advantages in preserving spatial heterogeneity and structural realism. The proposed EdgeGeoDiff framework further enhanced these properties through residual learning and frequency-aware guidance. In particular, the two-stage high-frequency enhancement strategy allows the model to progressively refine fine-scale structures, mitigating the tendency of diffusion models to over-smooth localized precipitation features.

A key observation from the quantitative and qualitative evaluations is that improvements in high-frequency reconstruction do not always correspond to monotonic gains in threshold-based metrics such as the CSI. This behavior reflects an inherent trade-off between structural fidelity and detection performance. Enhanced edge sharpness and local gradients may increase false alarms under fixed thresholds, even when the reconstructed structures are more physically realistic. Similar phenomena have been reported in previous precipitation downscaling studies, suggesting that evaluation metrics should be interpreted in conjunction with structural and frequency-domain analyses rather than in isolation.

The ablation studies further revealed that the effectiveness of EdgeGeoDiff arose from the coordinated interaction of multiple design components. Residual learning reduced the skewness and dynamic range of the diffusion target, stabilizing the learning process and facilitating high-frequency correction. Geographical prior encoding provides large-scale spatial constraints that improve consistency, while edge-aware feature interaction selectively reinforces structure-related high-frequency components. Importantly, intermediate configurations may yield higher CSI values due to smoother outputs, but the full model achieves a more balanced trade-off across reconstruction accuracy, structural coherence, and detection reliability.

Despite these advantages, several limitations remain. The model showed reduced sensitivity to extreme precipitation in some cases, which appears to be influenced by both the residual formulation and the initial upsampling stage in the two-stage framework. In addition, the use of geographical priors introduces region-specific assumptions that may limit direct generalization to datasets with different climatic or topographic characteristics. These limitations highlight opportunities for further methodological refinement.

Overall, this work suggests that diffusion-based downscaling, when combined with appropriate structural guidance, provides a viable and interpretable alternative to purely deterministic super-resolution methods for precipitation reconstruction. Emphasizing frequency-domain behavior and structural consistency offers a more comprehensive evaluation paradigm for geophysical downscaling tasks.

## 6. Conclusions

In this paper, we presented EdgeGeoDiff, a diffusion-based framework for precipitation downscaling that focuses on physically meaningful high-frequency reconstruction and structural consistency. The proposed method integrates a residual formulation to alleviate target distribution skewness, a two-stage high-frequency enhancement strategy to progressively refine fine-scale structures, and auxiliary geographic and edge-aware guidance to constrain spatial coherence.

Comprehensive experiments demonstrated that EdgeGeoDiff consistently outperformed representative single-image super-resolution and diffusion-based baselines, including ResDiff, particularly in terms of frequency-domain consistency, boundary clarity, and detection-oriented metrics. The results indicate that incorporating physically meaningful geographic factors and structural guidance helps the model better capture the spatial heterogeneity of precipitation fields. Although diffusion models are not currently the dominant paradigm for conventional super-resolution benchmarks, our findings suggest that they remain well suited for precipitation downscaling tasks where structural fidelity and spatial consistency are critical.

Future work will focus on improving the representation of extreme precipitation events through adaptive upsampling strategies and precipitation-aware diffusion guidance. In addition, relaxing or reducing the dependence on geographic prior constraints may further enhance the generalization ability of the framework for broader natural precipitation datasets. Since the current framework is primarily designed for precipitation, extending the proposed methodology to other climate variables, such as temperature and wind, will also be explored to evaluate its broader applicability in climate data downscaling.

## Figures and Tables

**Table 1:** Quantitative results of precipitation downscaling with a 4× scaling factor. The best results are highlighted in bold.

Method	PSNR↑	RMSE↓	SSIM↑	CSI↑	PSD-MSE↓
Bicubic	46.16	0.656	0.9697	0.921	$3.45\times 10^{-6}$
EDSR	51.62	0.322	0.9920	0.910	$5.60\times 10^{-5}$
SRGAN	50.07	0.375	0.9890	0.856	$8.00\times 10^{-6}$
Sr3	51.97	0.317	0.9916	0.940	$1.93\times 10^{-5}$
ResDiff	51.82	0.330	0.9921	0.928	$1.17\times 10^{-6}$
EdgeGeoDiff	**52.04**	**0.312**	**0.9922**	**0.956**	$2.86\times 10^{-5}$

**Table 2:** CSI scores under different precipitation thresholds. The best results are highlighted in bold.

Method	0.1	0.5	1	5	10
Bicubic	0.921	0.866	0.814	0.565	0.356
EDSR	0.901	0.903	0.882	0.708	0.525
SRGAN	0.856	0.876	0.861	0.689	0.482
Sr3	0.940	0.924	0.893	0.710	0.528
ResDiff	0.928	0.921	0.894	0.712	0.520
EdgeGeoDiff	**0.956**	**0.933**	**0.896**	**0.719**	**0.537**

**Table 3:** PSD-MSE comparison of different models after Gaussian high-frequency filtering. The best results are highlighted in bold.

λ	5	10	15
Bicubic	$8.55\times 10^{-5}$	$3.89\times 10^{-5}$	$2.31\times 10^{-5}$
SRGAN	$1.92\times 10^{-4}$	$1.02\times 10^{-4}$	$1.76\times 10^{-5}$
Sr3	$3.19\times 10^{-5}$	$1.51\times 10^{-5}$	$7.00\times 10^{-6}$
ResDiff	$2.60\times 10^{-5}$	$1.34\times 10^{-5}$	$4.87\times 10^{-6}$
EdgeGeoDiff(ours)	$2.57\times 10^{-5}$	$1.30\times 10^{-6}$	$4.22\times 10^{-6}$

**Table 4:** Band-wise structural similarity (SSIM) for frequency-domain evaluation. The best results are highlighted in bold.

Method	0.0–0.3	0.3–0.6	0.6–1.0
Bicubic	0.8580	0.8162	0.8204
Sr3	0.9104	0.8837	0.8625
ResDiff	**0.9497**	**0.9066**	0.8759
EdgeGeoDiff(ours)	0.9262	0.9064	**0.8826**

**Table 5:** The quantitative results of different geographic factors on downscaling results is discussed. * indicates the added geographic factor, surface roughness. The best results are highlighted in bold.

Geographic Factor	PSNR↑	RMSE↓	CSI↑
5-factor	**52.04**	**0.312**	**0.956**
None	51.63	0.330	0.890
1-factor (elevation)	51.71	0.329	0.932
3-factor	51.76	0.329	0.940
6-factor	51.30	0.341	0.941
6-factor *	51.70	0.329	0.901
7-factor	51.99	0.319	0.910

**Table 6:** Statistical relationship between precipitation residuals and topographic variables. Random forest importance reflects nonlinear contributions, while Pearson correlation and mutual information measure linear and general statistical dependence, respectively.

Geographical Factors	RF Importance	Pearson *r*
elevation	0.1970	−0.0047
Slope	0.1182	−0.0025
Slope gradient	0.1454	−0.0007
Aspect	0.1618	0.0001
Aspect gradient	0.1438	0.0003
Surface roughness	0.1163	0.0020
Topographic relief	0.1175	−0.0025

**Table 7:** Ablation study of different setting. The best results are highlighted in bold.

pre-HFAN	GPE	EAFIM	PSNR↑	RMSE↓	CSI↑
✓	✓	✓	**52.04**	**0.312**	**0.956**
✓	✓		51.62	0.333	0.911
✓		✓	51.63	0.330	0.890
✓			51.58	0.332	0.947

**Table 8:** Ablation study of residual structure. The best results are highlighted in bold.

Method	PSNR↑	RMSE↓	SSIM↑
Non-Residual	51.62	0.333	0.911
EdgeGeoDiff (ours)	**52.04**	**0.312**	**0.956**

## Data Availability

The datasets analyzed during the current study are not publicly available due to data usage agreements but are available from the corresponding author upon reasonable request. The implementation code is publicly available at https://github.com/lieyandadao/EdgeGeoDiff (accessed on 9 March 2026).
